# The Role of Personal Biological Resource in the Job Demands-Control-Support Model: Evidence From Stress Reactivity

**DOI:** 10.3389/fpsyg.2021.658180

**Published:** 2021-06-02

**Authors:** Huihua Deng, Yuli Zhuo, Xingliang Qi, Hanyao Wu, Yapeng Liu, Jianmei Li, Caixiang Jin

**Affiliations:** ^1^Key Laboratory of Child Development and Learning Science (Southeast University), Ministry of Education, Nanjing, China; ^2^Institute of Child Development and Education, Southeast University, Nanjing, China; ^3^Department of Medical Humanity, School of Humanities, Southeast University, Nanjing, China; ^4^College of Pro-School Education, Nanjing Xiaozhuang University, Nanjing, China; ^5^Office of Social Science, Southeast University, Nanjing, China; ^6^Department of Nursing, Nanjing Integrated Traditional Chinese and Western Medicine Hospital, Nanjing, China

**Keywords:** psychological demands, job control, social support, job burnouts, stress reactivity, hair cortisol content, hospital nurses

## Abstract

Job resources can buffer the deleterious effect of adverse work environments. Extant studies on the interaction pattern between job resources and adverse environments were confined to the diathesis stress model. This traditional perspective has received the challenge from the differential susceptibility model and the vantage sensitivity model. Additionally, stress reactivity may be one of the important job resources at the personal biological level, but its moderating role was short of empirical research. This study aimed to examine how stress reactivity interacts with work environments in predicting job burnouts among 341 Chinese hospital female nurses. This study selected job control and job support representative of supportive environments and psychological demands representative of an adverse environment and the cortisol content in 1-cm hair segment as a biomarker to assess individual’s stress reactivity in 1 month. The nurses self-reported their work environments and job burnouts and provided 1-cm hair segments closest to the scalp. Hair cortisol content was measured with high-performance liquid chromatography-tandem mass spectrometry. The interaction pattern was examined with multiple linear regressions and the analysis of region of significance (RoS). The regression revealed that the interaction of hair cortisol content with job control could positively predict professional efficiency among nurses, with psychological demands could negatively predict emotional exhaustion, and with coworker support could negatively predict professional efficiency. The RoS analysis revealed that nurses with high cortisol levels had not only significantly higher professional efficiency than those with low cortisol levels in high job control but also significantly lower professional efficiency in low job control. Nurses with high cortisol levels had significantly higher emotional exhaustion than those with low cortisol levels in low psychological demands. Nurses with low cortisol levels had not only significantly higher professional efficiency than those with high cortisol levels in high coworker support but also significantly lower professional efficiency in low coworker support. The interaction patterns of stress reactivity with both job control and coworker support were consistent with the differential susceptibility model, but the interaction between stress reactivity and psychological demands supported the vantage sensitivity model.

## Introduction

Numerous studies have well documented that adverse work environments can have detrimental effects on the employee’s well-being, health, and work-related outcomes (Salvagioni et al., 2017). Among various outcomes, job burnouts are the typical fatigue and exhaustion syndromes that are elicited by adverse work environments, such as high job demands (Demerouti et al., 2001; Halbesleben and Buckley, 2004). The deleterious effect of high job demands is considered to be buffered by sufficient job resources as emphasized in the job demands-resources (JDR) model (Bakker and Demerouti, 2007) and high job control and more social support as emphasized in the job demands-control (JDC) model (Karasek, 1979; van der Doef and Maes, 1998) and the job demands-control-support (JDCS) model (Johnson and Hall, 1988; van der Doef and Maes, 1998). That is, not all employees are to the same extent susceptible to the deleterious influences of high job demands. For example, employees with more job resources have been considered to be more resistant to stress exposure in the workplace than their colleagues with fewer job resources, showing lower levels of adverse outcomes in the identical adverse work environment (Bakker et al., 2005). However, most extant empirical studies focused on the buffering role of the external organizational resources related to job characteristics in the workplace, such as social support from coworkers and supervisors, work autonomy, quality of the relationship with the supervisor, and performance feedback (Demerouti et al., 2001; Bakker et al., 2003, 2005; Johnson and Spector, 2007). Comparatively limited studies had examined the buffering role of personal mental resources, such as optimism, extraversion, compassion satisfaction, dispositional punishment and reward sensitivity, organization-based self-esteem, emotional intelligence, self-efficacy, self-control capacity, political skill, and recovery experience as resource replenishment (Jex and Elacqua, 1999; Van Yperen and Snijders, 2000; Mkikangas and Kinnunen, 2003; Johnson and Spector, 2007; Chi et al., 2011; Pugh et al., 2011; Tremblay and Messervey, 2011; Schreurs et al., 2014; Diestel et al., 2015; Gu and You, 2019; Usman et al., 2020). To date, there are a few studies exploring the buffering effect of personal biological resources, such as sleep quality (Diestel et al., 2015) and stress reactivity (Deng et al., 2020).

On the other hand, regarding the buffering role of job resources in adverse work environments, extant studies focused on the interaction showing the pattern that is consistent with the diathesis stress model, such as the interaction between fewer job resources and highly stressful work environments (Bakker et al., 2005). This traditional focus has received the challenge from other interaction patterns as outlined in the differential susceptibility model and the vantage sensitivity model, which were developed by Belsky et al. (Belsky et al., 2007; Belsky and Pluess, 2009; Ellis et al., 2011; Pluess and Belsky, 2013) for explaining children’s susceptibility to environmental influences. However, there are a few studies exploring the interaction patterns that follow the two novel models. Additionally, previous studies were mostly confined to the adverse and pathogenic responses (e.g., emotional exhaustion) to adverse work environments under the frame of the JDC model or the JDCS model (Johnson and Hall, 1988; van der Doef and Maes, 1998) and the JDR model (Bakker and Demerouti, 2007). Notably, the presence of adverse environments and negative work-related outcomes is not equivalent to the absence of supportive environments and adaptive outcomes in work environments. However, there are few studies addressing the adaptive work-related response of individuals with high-risk traits (or fewer protective factors) in a supportive work environment with high job controllability or more social support, which may be supplied by the organization through the interior reform, such as adjustment on employee’s working position. Currently unknown is whether the work-related responses to a supportive environment differ in individuals with more and fewer protective factors, especially for job resources at the personal biological level (i.e., stress reactivity here). Therefore, the current study aimed to examine which model the interaction patterns between work environments and stress reactivity as personal biological resources supports across adverse and supportive environments and across maladaptive and adaptive outcomes for fully understanding the importance of the biological processes in the employee’s responses to work environments.

## Background

### Stress Reactivity as Personal Biological Resources

As protective factors, job resources refer to those physical, psychological, social, or organizational aspects of the job that may be functional in achieving work goals, or reduce job demands at the associated physiological and psychological costs, or stimulate personal growth and development (Demerouti et al., 2001). Job resources contain external resources (e.g., organizational and social resources) and internal personal resources (Xanthopoulou et al., 2007). Personal resources that are closely related to resiliency belong to aspects of the self at the cognitive level, emotional level, and biological level including individuals’ sense of their ability to control and impact upon their environment successfully (Hobfoll, 2002; Hobfoll et al., 2003), such as cognitive features and action patterns. Stress reactivity may be one of the important personal biological resources, with high level indicative of high susceptibility to the influences of work environment because it has been conceptualized as a highly biological sensitivity to context (Boyce and Ellis, 2005). The hypothalamic-pituitary-adrenal (HPA) axis is one of the stress-sensitive nervous systems that help organs adapt to stressful events (Spiga et al., 2014). The activity of the HPA axis reflects individual’s stress reactivity (Obradovi et al., 2010). Recently, a pilot study has demonstrated that the HPA activity (or stress reactivity) as personal biological resources can play a moderating role as personal mental resources do (Deng et al., 2020). However, the recent study only investigated the interaction of the HPA activity (or stress reactivity) with emotional labor (a special aspect of job demands) in predicting job burnouts. Therefore, it needs to extend the moderating role of stress reactivity to more generalized stressful work environments from other aspects of high job demands or low job controllability or less social support.

### Three Models on the Interaction Patterns

The diathesis stress model suggests that in adverse environments, individuals with high-risk traits (e.g., insufficient personal resources, risk gene, and higher stress reactivity) display higher vulnerability than those with low-risk traits, being more liable to develop various maladaptive outcomes (e.g., emotional exhaustion), but there are no differences between the two groups in supportive environments (Belsky and Pluess, 2009). The differential susceptibility model proposes that individuals with certain traits will be more vulnerable to an adverse environment, following the worse outcomes, but benefit from a supportive environment, resulting in better outcomes (Belsky and Pluess, 2009). The vantage sensitivity model posits that individuals with vantage sensitivity characteristics display better adaptation in a supportive environment than the other individuals because they are likely to be more sensitive to the positive contexts, but there are no differences between the two groups in an adverse environment (Pluess and Belsky, 2013). The patterns of the three interaction models can be elucidated with the analysis of region of significance (RoS) and the proportion of interaction (PoI) index and the proportion affected (PA) index (Roisman et al., 2012). However, the interaction pattern between personal biological resources and work environments was not further confirmed with the analysis of RoS in a recent study demonstrating a moderating role of stress reactivity as personal biological resources in the relationship between stress reactivity and emotional labor (Deng et al., 2020). Therefore, it needs to follow the approach strategy and to further elucidate the interaction pattern between stress reactivity and work environments in terms of the analysis of RoS in combination with the PoI index and the PA index.

## The Present Study

The present study aimed to examine how work environments interact with stress reactivity in predicting work-related outcomes. Under the frame of the JDCS model, psychological demands as the existence of socially “objective” environments is representative of an adverse environment, and worker’s decision latitude (or job control) and social support at the work environment are representative of supportive environments (Johnson and Hall, 1988; Xie, 1996; Karasek et al., 1998). Therefore, this study selected psychological demands, job control, and job support as work environmental factors, emotional exhaustion and depersonalization as examples of negative outcomes, and professional efficiency as an example of positive outcome. The cortisol content in 1-cm hair segment was utilized as a biomarker to assess the individual’s stress reactivity in 1 month, which is the period that most psychological measurements cover. This is because cortisol as an end product of the HPA axis is a biomarker of the HPA activity (Spiga et al., 2014) and also one of the reliable biomarkers for assessing an individual’s stress reactivity (Obradovi et al., 2010). Salivary cortisol was used to estimate an individual’s stress reactivity (Obradovi et al., 2010, 2015). If the growth rate of hair shaft is 1 cm per month, the cortisol content in the 1-cm hair segment would reliably reflect the HPA activity over 1 month or the accumulative reactivity to daily stressful events over 1 month (Russell et al., 2012) compared with salivary and urinary cortisol levels, reflecting an acute and a short-term activity of the HPA axis over several minutes, several hours, and up to 1 day (Dickerson and Kemeny, 2004). This study focused on Chinese hospital nurses who suffered from high job stresses and job burnouts and had relatively low job satisfaction (Wu et al., 2018).

Previous studies had demonstrated that stress reactivity could moderate the relationship between environmental factors and adolescents’ psychological adaptations (Wiel et al., 2004; Obradovi et al., 2010, 2015; Owens et al., 2018; Xu et al., 2019) and the relationship between emotional labor and job burnouts (Deng et al., 2020). Thus, we hypothesized that hair cortisol levels (or stress reactivity) could moderate the relationship between environmental factors and work-related outcomes among Chinese nurses. Moreover, we proposed three alternative hypotheses based on the aforementioned three models. From the perspective of the differential susceptibility model, we expected that nurses with high hair cortisol levels (or high stress activity) might be more vulnerable to adverse environments (e.g., highly stressful demands, low controllability, and less support) but benefit from supportive environments (e.g., low stressful job demands, high job controllability, and more support) because of their high susceptibility to the influences of work environment. In other words, high hair cortisol level was a plastic factor (i.e., it was not only a risk factor in the context of adverse environments but also a promoting factor in supportive environments). From the vantage sensitivity perspective, we expected that nurses with high hair cortisol levels might display better adaptation in a supportive environment than those with a low stress reactivity, but there was no difference in adverse environments between the two groups, or that high hair cortisol level was a vantage sensitivity characteristic. From the diathesis stress perspective, we expected that nurses with high hair cortisol levels might suffer from more maladaptation in adverse environments than those with low hair cortisol levels, but there was no difference in supportive environments between them, or that high stress reactivity was a risk factor.

## Materials and Methods

### Participants

This study recruited female nurses through posting the advertisement in Tencent’s WeChat and QQ groups. Initially, 495 female nurses were randomly recruited during September 2017–January 2018 from the nine hospitals that are near our university in Nanjing city, China. All participants provided written informed consent before inclusion. Among them, 452 nurses (91.31%) completed the questionnaires including demographic information, job content questionnaire, job burnouts, and job satisfactions, and 408 nurses (82.42%) provided their hair strands and the hair-related information. Here, 67 participants (13.54%) were excluded. The exclusion criteria were obese (body mass indexes ≥30); alcoholics; smokers; shorter hair strands (<1 cm); dyed, permed, or bleached hair; diseases (e.g., canker sores and inflammation); or medicine intake (e.g., glucocorticoid and antibiotics drugs), which might influence the contents of cortisol in hair (Wosu et al., 2013). Finally, 341 nurses (68.89%) participated in the present study. This study followed the Declaration of Helsinki and was approved by the Health Science Research Ethics Board of Southeast University.

The 341 nurses worked in different types of departments: emergency department (32.55%), intensive care unit (ICU; 15.25%), emergency intensive care unit (EICU; 7.62%), rehabilitation department (12.02%), radiotherapy department (7.92%), department of medical psychology (8.80%), and others (15.84%) including struma department, pediatrics, orthopedics, neurology, neurosurgery, internal medicine, endocrine department, dental department, Chinese medicine surgery, and operating theater over the past 1 year. Of those, 48.99% nurses served as a nurse for less than 5 years, 32.26% for 5–15 years, and 18.77% over 15 years. Among them, 31.96% nurses were junior nurses, 46.92% were senior nurses, and 21.11% were charge nurses and over. Income of 3.81% nurses was less than 2,000 RMB per month, 21.11% earned between 2,001 and 3,000 RMB per month, 52.20% earned between 3,001 and 5,000 RMB per month, and 22.87% earned over 5,001 RMB per month. In addition, 90.03% nurses were in the 8-h three-shift scheduling and 9.97% nurses were in the 12-h two-shift scheduling.

### Procedures

The participants self-reported their demographic information including nurses’ working department, professional titles, working duration as a nurse, the monthly income and shift scheduling pattern, and their psychological states including job characteristics and job burnouts over the past 1 month. Two weeks after the questionnaire’s collection, hair samples (over 20 mg in weight) were collected by the well-trained research assistants to match the psychological data in time span. The delay in hair sampling was recommended by LeBeau et al. (2011), who thought that 1–3 mm of the hair strands are embedded in the skin and the 1–2-mm hair strands closest to the scalp cannot be completely cut with scissors if the hair growth rate is 1 cm per month (Pragst and Balikova, 2006). The collected hair samples were sealed with foil to avoid direct irradiation from sunlight and then were stored in a dry and dark environment at room temperature until the analysis.

### Measures

#### Psychological and Social Characteristics of Jobs

The job characteristics were measured with subscales of five-item psychological demands, nine-item job control (or decision latitude), four-item supervisor support, and four-item coworker support in the job content questionnaire (JCQ) developed by Karasek (1985) and translated into the Chinese version by Cheng et al. (2003). Job control subscale consists of six-item skill discretion, such as “My job requires that I learn new things” and three-item decision authority, such as “My job allows me to make a lot of decision on my own.” The sample item in psychological demands is “My job requires working very fast,” in supervisor support is “My supervisor pays attention to what I am saying,” and in coworker support is “People I work with are friendly.” Each item is rated on a four-point Likert scale ranging from 1 (strongly disagree) to 4 (strongly agree). Each scale was estimated with a sum of weighted item scores according to the user’s guide (Karasek, 1985), higher scores indicating higher stress for psychological demands, but lower scores indicating higher stress for the other three job characteristics. The scale was proven to have good reliability and validity in Chinese workers (Xie, 1996; Cheng et al., 2003). In the present study, the Cronbach’s alpha coefficient was 0.70, 0.81, 0.85, and 0.87 for the four subscales.

#### Job Burnout

Job burnout was measured with Maslach Burnout Inventory-General Survey (MBI) developed by Schaufeli et al. (1996) and translated into Chinese by Li and Shi (2003). The Chinese version of MBI includes 16 items assessing the frequency of nurses’ experiencing burnout and consists of three subscales measuring emotional exhaustion (five items), depersonalization (five items), and professional efficiency (six items). Each item is rated on a 7-point Likert scale ranging from 1 (never) to 7 (always), higher scores indicating heavier burnout for emotional exhaustion and depersonalization but lower scores indicating heavier burnout for professional efficiency. The scale was proven to have good reliability and validity in Chinese workers (Li and Shi, 2003). In the present study, average score for each subscale was utilized, and the Cronbach’s alpha coefficient was 0.95, 0.82, and 0.86 for the three subscales.

#### The Analysis of Hair Cortisol Contents

The detailed procedures of analyzing hair cortisol contents (HCCs) were described elsewhere (Chen et al., 2019). Briefly, the 1-cm hair strands closest to the scalp were treated by a standard protocol: washing with methanol, cutting into pieces, incubation in methanol, centrifugation, solid-phase extraction, and drying with pure nitrogen gas. The dried residue was redissolved in 50-μl methanol for cortisol analysis that was done on a Qtrap 3200 liquid chromatography-tandem mass spectrometer (ABI, United States). Cortisol was ionized with an atmosphere pressure chemical ionization and identified in positive ion mode using multiple reactions monitoring mode. The assay method had good linearity in the range of 0.8–250.0 pg/mg, showing the square coefficient of correlation at more than 0.99. It also had good sensitivity, accuracy, and precision, showing limits of detection and quantitation at 0.3 and 0.8 pg/mg, intra-day and inter-day coefficients of variation less than 15%, and recovery ranging between 85 and 115% (Chen et al., 2019), which fit the requirements of hair cortisol measurement.

### Data Preparation and Analysis Procedures

Prior to analyses, all variables were examined for accuracy of data entry, missing data, data normality, and common method bias. Data were analyzed by the statistical package SPSS 22.0 for Windows. Confirmatory factor analysis was performed by Lisrel 8.70. Percentages of missing data were less than 1.0% for all the predictive and outcome variables, and there were no missing data for the moderating variable. Missing data for all the predictive and outcome variables were handled using the expectation-maximization algorithm (Schafer and Graham, 2002). The data distribution normality was examined with one-sample Kolmogorov-Smirnov test. HCC showed non-normal distribution (*p* < 0.001) with a skewness at 5.636 ± 0.132 and a kurtosis at 40.683 ± 0.263 and became normally distributed (*p* = 0.200) with a skewness at 0.130 ± 0.132 and a kurtosis at 1.139 ± 0.263 after a log-transformation that could effectively reduce the skewness and kurtosis. The log-transformed HCC data were used for the next analyses including Pearson’s correlation and the hierarchical multiple regression.

## Results

### Descriptive Statistics

Harman’s single-factor test was performed to assess the common method variance biases (Podsakoff et al., 2003). The items on job control and emotional exhaustion did not generate the unique factor with the explained variance more than 40% (30.32% as examined with an exploratory factor analysis through principal components extraction) and did not converge on a single factor [*χ*^2^/df = 8.138, goodness-of-fit index (GFI) = 0.741, comparative fit index (CFI) = 0.755, Tucker-Lewis index (TLI) = 0.731, root mean square error of approximation (RMSEA) = 0.145 as tested with a confirmatory factor analysis]. Similarly, the items on the other predictors and the outcome variables did not generate a single factor. All the items on the four job characteristics and job burnouts also did not generate a single factor. It was thus assumed that the common method variance bias was not serious in this study.

As listed in Table 1, job control and supervisor support were significantly and negatively correlated with emotional exhaustion and depersonalization (*p*s < 0.05) and positively correlated with professional efficiency (*p*s < 0.05). Psychological demands were significantly and positively correlated with emotional exhaustion and depersonalization (*p*s < 0.05) but not with professional efficiency (*p* > 0.05). Coworker support was significantly and negatively correlated with depersonalization (*p* < 0.05) and positively with professional efficiency (*p* < 0.05) but not with emotional exhaustion (*p* > 0.05). HCC was not correlated with job characteristics and job burnouts (*p*s > 0.05). Additionally, work duration as a nurse was significantly and negatively correlated with supervisor support and coworker support (*r* = −0.189, *p* < 0.001 and *r* = −0.172, *p* = 0.001) and positively with professional efficiency (*r* = 0.152, *p* = 0.005) but not with job control, psychological demands, HCC, and the other burnouts (*p*s > 0.115). There were significant differences among different working departments in psychological demands, emotional exhaustion, and depersonalization (*F*_6, 334_ = 9.168, *p* < 0.001; *F*_6, 334_ = 6.642, *p* < 0.001; *F*_6, 334_ = 4.879, *p* < 0.001) but no differences in the other job characteristics, HCC, and professional efficiency (*p*s > 0.119). There were significant differences between two shift schedule patterns in psychological demands, emotional exhaustion, and depersonalization (*F*_1, 339_ = 4.834, *p* = 0.029; *F*_1, 339_ = 5.201, *p* = 0.023; *F*_1, 339_ = 4.318, *p* = 0.038) but no differences in the other job characteristics, HCC, and professional efficiency (*p*s > 0.250).

**Table 1:** Means, standard deviations, and Pearson correlation coefficients for job characteristics, hair cortisol content, and job burnouts (*n* = 341).

S. No.		1	2	3	4	5	6	7	8
1.	Job control	-							
2.	Psychological demands	−0.202^**^	-						
3.	Supervisor support	0.404^**^	−0.075	-					
4.	Coworker support	0.268^**^	0.061	0.510^**^	-				
5.	HCC^a^	0.079	−0.040	0.039	0.000	-			
6.	Emotional exhaustion	−0.250^**^	0.470^**^	−0.147^**^	−0.089	−0.045	-		
7.	Depersonalization	−0.315^**^	0.298^**^	−0.223^**^	−0.179^**^	−0.040	0.658^**^	-	
8.	Professional efficiency	0.240^**^	−0.062	0.116^*^	0.120^*^	0.034	−0.034	−0.163^**^	-
	*M*^b^	62.38^c^	33.69^d^	11.84	12.51	3.3	3.42	2.45	4.12
	*SD*^b^	8.38^c^	6.93^d^	1.84	1.50	0.3–49.6	1.51	1.33	1.24

* *p* < 0.05;

** *p* < 0.01;

*** *p* < 0.001.

a HCC refers to hair cortisol content. HCC is log-transformed for Pearson correlation analysis.

b HCC is presented as median and range (pg/mg) because HCC showed non-normal distribution and the other variables are presented as M and SD, where M is mean and SD is standard deviation.

c The score of job control in the next regression analysis was calculated as a standard score according to the formula, standard score = (the actual score−24)/(96–24).

d The score of job control in the next regression analysis was calculated as a standard score according to the formula, standard score = (the actual score−12)/(48–12).

### The Interaction Patterns Between Job Characteristics and Hair Cortisol Content

A total of 12 four-step moderated hierarchical regressions were conducted to examine the interactive effects between job characteristics and HCC on job burnouts. Demographic variables (i.e., working department, shift schedule pattern, and working duration as a nurse) were entered into the regression at Step 1. Job characteristics as the predictor were separately entered into the equation at Step 2. HCC as the moderator was entered at Step 3. Lastly, the interaction term between job characteristics and HCC was separately entered at Step 4. The amount of an additional explained variance was estimated after each step. Prior to the regression analyses, the moderator and all the independent variables except for type variables were centralized to effectively reduce multicollinearity (Aiken and West, 1991). The present 12 models had no serious collinearity as examined by the collinearity diagnoses where the tolerance was more than 0.2 and variance inflation factor was less than 5 for all the regression equations (Fox, 1991). In order to further explore the nature and directionality of the significant interactions, the simple slopes analyses in which the effects of higher and lower levels (i.e., 1 *SD* above the mean and 1 *SD* below the mean, *M*+1 *SD* and *M*−1 *SD*) of job characteristics were done in female nurses with higher and lower HCC levels (i.e., *M*+1 *SD* and *M*−1 *SD*) were performed according to the procedures proposed by Aiken and West (1991). The RoS approach was conducted to elucidate whether the interaction between job characteristics and HCC follows the differential susceptibility model or the vantage sensitivity model. The RoS was calculated from −2 SD to +2 SD from the means of job characteristics in terms of the probing interaction procedure developed by Hayes and Matthes (2009). The interaction patterns are determined according to the criteria based on RoS, PoI index, and PA index (Roisman et al., 2012).

The regression analysis revealed that job control was negatively associated with emotional exhaustion and depersonalization (*p*s < 0.001) and positively with professional efficiency (*p* < 0.001) as listed in Table 2. Psychological demands were positively associated with emotional exhaustion and depersonalization (*p*s < 0.001), but not with professional efficiency (*p* > 0.05) as listed in Table 3. Supervisor support was negatively associated with emotional exhaustion and depersonalization (*p* < 0.01 and *p* < 0.001) and positively associated with professional efficiency (*p* < 0.01) as listed in Table 4. Coworker support was negatively associated with depersonalization (*p* < 0.001) and positively associated with professional efficiency (*p* < 0.01), but not with emotional exhaustion (*p* > 0.05) as listed in Table 5. HCC was not associated with job burnouts (*p*s > 0.05). Additionally, Fisher *Z* test revealed that emotional exhaustion was more sensitive to psychological demands than depersonalization and professional efficiency (*Z* = 2.512 and *Z* = 5.109, *p*s < 0.05) and depersonalization than professional efficiency (*Z* = 2.597, *p* < 0.05). Among the three burnout syndromes, there were no differences in the sensitivity to the other three predictors, job control, coworker support, and supervisor support (*p*s > 0.05).

**Table 2:** Multiple linear regression results of job control, hair cortisol content, and their interaction against job burnouts (*n* = 341).

	Independent variable	Emotional exhaustion				Depersonalization				Professional efficiency			
	Predictive variable	Δ*R*^2^	*β*	*B*	*SE*	Δ*R*^2^	*β*	*B*	*SE*	Δ*R*^2^	*β*	*B*	*SE*
Step 1	Demographic variables^a^	0.111^***^				0.084^***^				0.045^d^			
	ICU		0.227^***^	0.955	0.265		0.244^***^	0.901	0.236		−0.162^*^	−0.556	0.224
	EICU		0.173^**^	0.983	0.345		0.174^**^	0.868	0.307		−0.068	−0.317	0.293
	Emergency department		0.322^***^	1.066	0.217		0.242^***^	0.703	0.193		−0.070	−0.189	0.184
	Radiotherapy department		0.039	0.251	0.360		−0.029	−0.162	0.321		−0.069	−0.363	0.306
	Psychology department		0.055	0.370	0.376		0.092	0.544	0.335		−0.061	−0.340	0.319
	Rehabilitation department		0.031	0.144	0.277		0.089	0.364	0.247		−0.032	−0.124	0.235
	Shift pattern		0.070	0.353	0.278		0.057	0.251	0.247		−0.008	−0.033	0.236
	Working duration		−0.026	−0.005	0.011		0.024	0.004	0.010		0.127^*^	0.022	0.010
Step 2	Job control	0.049^***^	−0.224^***^	−2.901	0.661	0.088^***^	−0.301^***^	−3.410	0.575	0.053^***^	0.233^***^	2.462	0.561
Step 3	HCC^b^	0.002	0.041	0.185	0.233	0.001	−0.034	−0.134	0.203	0.000	0.010	0.037	0.197
Step 4	JC × HCC^c^	0.001	0.031	1.049	1.722	0.001	−0.027	−0.783	1.500	0.012^*^	0.112^*^	3.055	1.059

*ΔR*^2^ is the change of R square, β is standardized regression coefficients, B is unstandardized regression coefficients, and SE is standard error of mean (the same below).

* *p* < 0.05;

** *p* < 0.01;

*** *p* < 0.001.

a Demographic variables include type variables, working department and shift pattern (i.e., the 8-h three-shift or 12-h two-shift scheduling pattern) and continuous variable (i.e., working duration as a nurse). Because working department containing seven types of departments was a dummy variable, intensive care unit (ICU), emergency intensive care unit (EICU), emergency department, radiotherapy department, department of medical psychology (psychology department), and rehabilitation department is coded as 1 in turns while the other departments as a reference is coded as 0. The 8-h three-shift and 12-h two-shift scheduling patterns are coded as 0 and 1, respectively.

b HCC refers to hair cortisol content.

c JC × HCC refers to the interaction between job control and hair cortisol content.

d *p* = 0.054.

**Table 3:** Multiple linear regression results of psychological demands, hair cortisol content, and their interaction against job burnouts (*n* = 341).

	Independent variable	Emotional exhaustion				Depersonalization				Professional efficiency			
	Predictive variable	Δ*R*^2^	*β*	*B*	*SE*	Δ*R*^2^	*β*	*B*	*SE*	Δ*R*^2^	*β*	*B*	*SE*
Step 1	Demographic variables^a^												
Step 2	PD^b^	0.145^***^	0.412^***^	4.328	0.540	0.049^***^	0.240^***^	2.207	0.511	0.002	−0.045	−0.387	0.500
Step 3	HCC^c^	0.003	0.054	0.246	0.219	0.001	−0.039	−0.156	0.207	0.001	0.025	0.092	0.203
Step 4	PD × HCC^d^	0.013^*^	−0.116^*^	−3.538	1.460	0.000	−0.016	−0.419	1.395	0.000	−0.004	−0.097	1.366

* *p* < 0.05;

*** *p* < 0.001.

a Demographic variables show the same results as Table 2.

b PD refers to psychological demands.

c HCC refers to hair cortisol content.

d PD × HCC refers to the interaction between psychological demands and hair cortisol contents.

**Table 4:** Multiple linear regression results of supervisor support, hair cortisol content, and their interaction against job burnouts (*n* = 341).

	Independent variable	Emotional exhaustion				Depersonalization				Professional efficiency			
	Predictive variable	Δ*R*^2^	*β*	*B*	*SE*	Δ*R*^2^	*β*	*B*	*SE*	Δ*R*^2^	*β*	*B*	*SE*
Step 1	Demographic variables^a^												
Step 2	Supervisor support	0.026^**^	−0.165^**^	−0.136	0.043	0.050^***^	−0.228^***^	−0.165	0.038	0.023^**^	0.157^**^	0.106	0.037
Step 3	HCC^b^	0.001	0.032	0.146	0.236	0.002	−0.045	−0.178	0.207	0.000	0.020	0.073	0.200
Step 4	SS × HCC^c^	0.000	−0.015	−0.045	0.155	0.006	−0.080	−0.205	0.136	0.003	−0.058	−0.138	0.132

** *p* < 0.01;

*** *p* < 0.001.

a Demographic variables show the same results as Table 2.

b HCC refers to hair cortisol content.

c SS × HCC refers to the interaction between supervisor support and hair cortisol content.

**Table 5:** Multiple linear regression results of coworker support, hair cortisol content, and their interaction against Maslach job burnouts (*n* = 341).

	Independent variable	Emotional exhaustion			Depersonalization					Professional efficiency			
	Predictive variable	Δ*R*^2^	*β*	*B*	*SE*	Δ*R*^2^	*β*	*B*	*SE*	Δ*R*^2^	*β*	*B*	*SE*
Step 1	Demographic variables^a^												
Step 2	Coworker support	0.007^d^	−0.088^d^	−0.089	0.053	0.030^***^	−0.176^***^	−0.156	0.047	0.022^**^	0.150^**^	0.124	0.045
Step 3	HCC^b^	0.001	0.024	0.108	0.238	0.003	−0.056	−0.221	0.209	0.001	0.027	0.100	0.200
Step 4	CS × HCC^c^	0.002	−0.042	−0.145	0.182	0.000	0.021	0.065	0.160	0.013^*^	−0.114^*^	−0.325	0.102

* *p* < 0.05;

** *p* < 0.01;

*** *p* < 0.001.

a Demographic variabl;s showed the same results as Table 2.

b HCC refers to hair cortisol content.

c CS × HCC refers to the interaction between coworker support and hair cortisol contents.

d *p* = 0.097.

**Figure 1:**
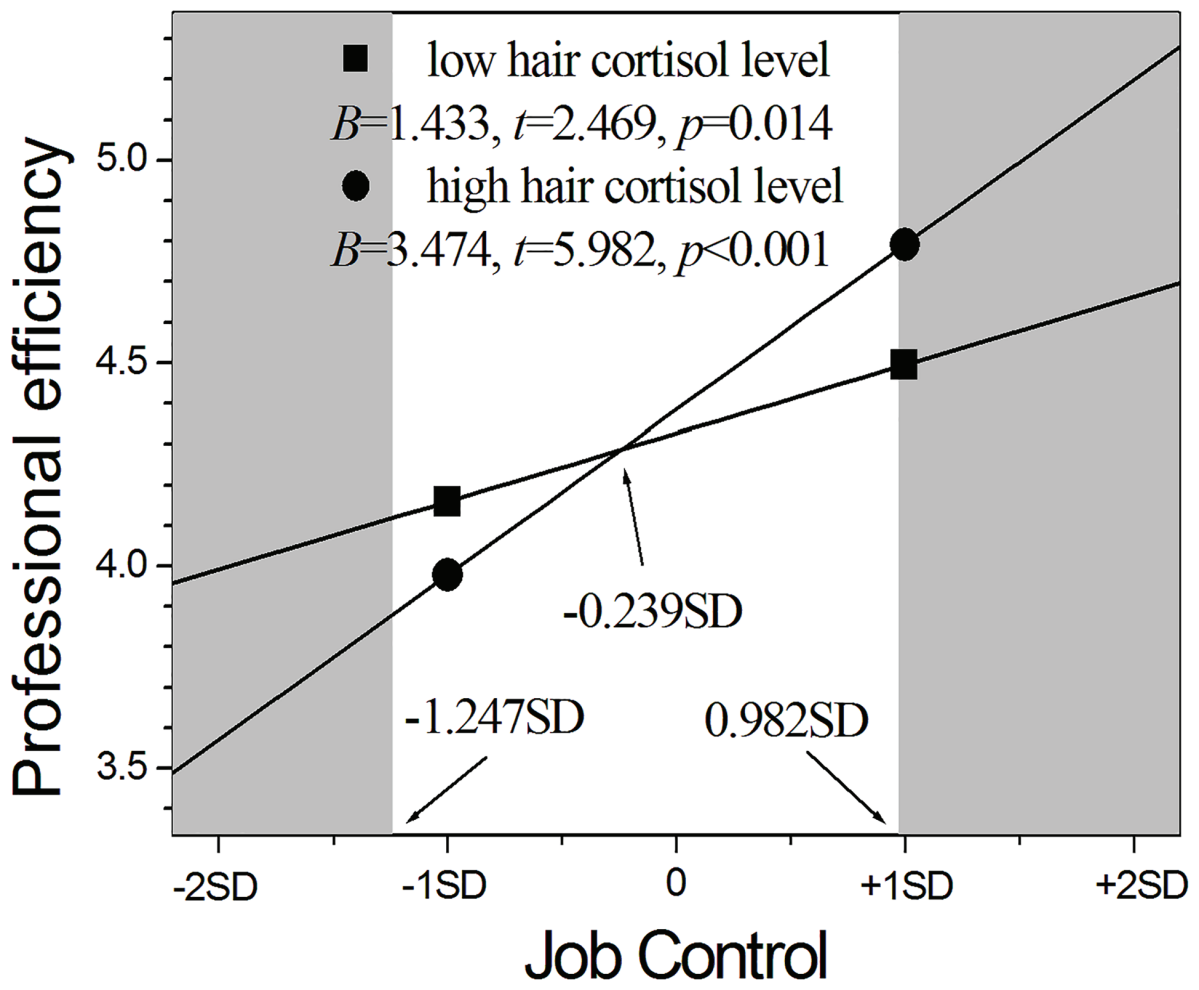
The moderating role of hair cortisol in the relationship between job control and professional efficiency.

Notably, the interaction of HCC with job control could positively predict professional efficiency (*p* < 0.05) as listed in Table 2, with psychological demands could negatively predict emotional exhaustion (*p* < 0.05) as listed in Table 3, and with coworker support could negatively predict professional efficiency (*p* < 0.05) as listed in Table 5. Moreover, the influence of job characteristics on nurses’ job burnouts varied across different HCC levels as demonstrated by simple slopes analyses. As shown in Figure 1, the positive influence of job control on professional efficiency was stronger in nurses with high cortisol levels than those with low cortisol levels (*B* = 3.474 vs. *B* = 1.433), although the influence was significant for nurses in both groups (*p* < 0.001 and *p* < 0.05). The RoS approach further revealed that nurses with high cortisol levels had significantly higher professional efficiency than those with low cortisol levels in the context of higher job control above 0.982 SD from the mean (see the right shaded area in Figure 1) and had significantly lower professional efficiency in the context of lower job control below −1.247 SD from the mean (see the left shaded area in Figure 1), but the two groups had no difference in the region between −1.247 and 0.982 SD from the mean of job control. The PoI index was 0.62, and the PA index was 0.51. These results indicated that the interaction between job control and HCC supports the differential susceptibility model.

**Figure 2:**
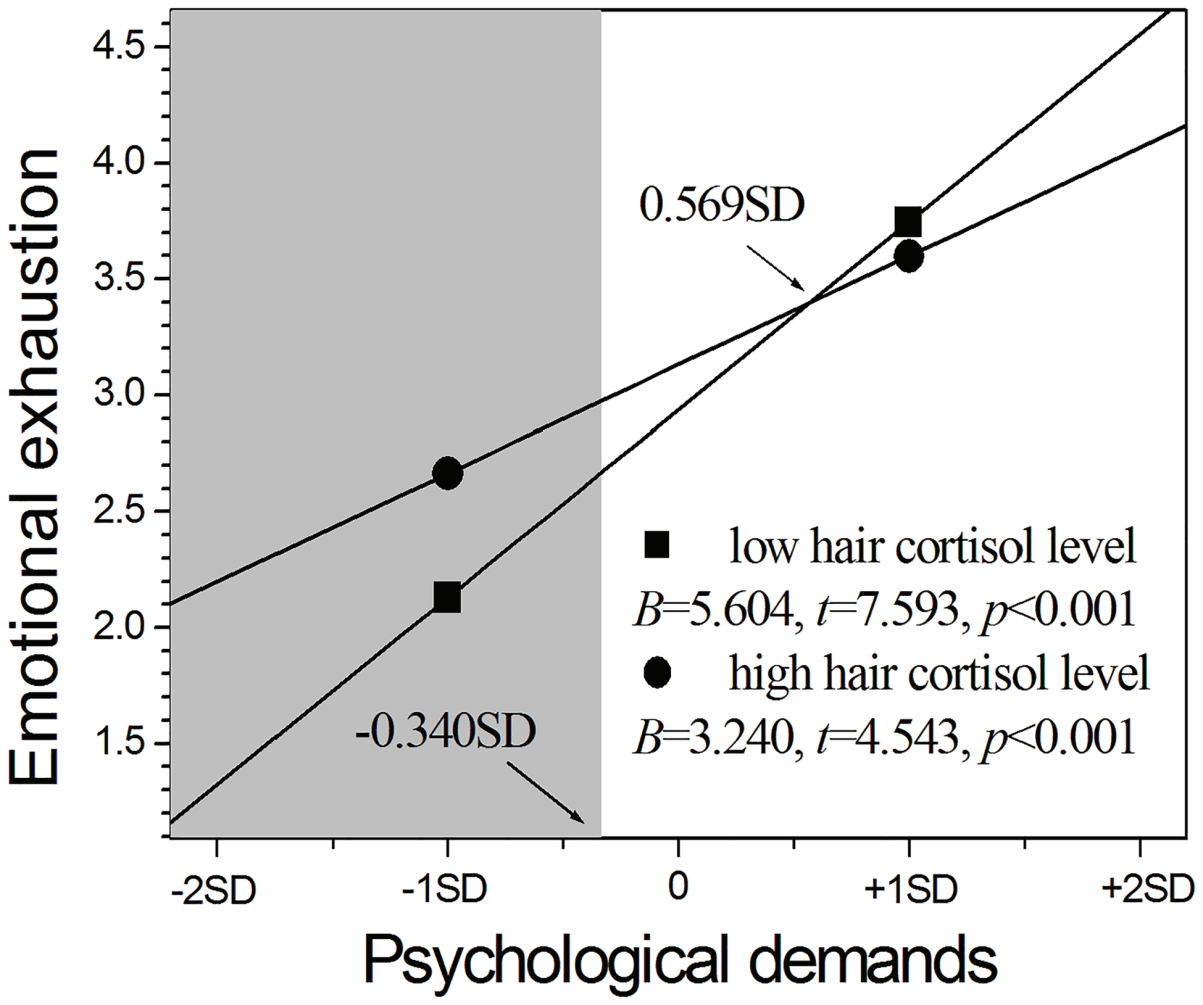
The moderating role of hair cortisol in the relationship between psychological demands and emotional exhaustion.

As shown in Figure 2, a weaker influence of psychological demands on emotional exhaustion was observed in nurses with high cortisol levels than those with low cortisol levels (*B* = 3.240 vs. *B* = 5.604), although the positive influence was significant for both groups (*p*s < 0.001). The RoS results further revealed that nurses with high cortisol levels had significantly higher emotional exhaustion than those with low cortisol levels in the context of lower psychological demands below −0.340 SD from the mean (see the left shaded area in Figure 2), but the two groups had no difference in the context of higher psychological demands above −0.340 SD until +2 SD from the mean of psychological demands. The PoI index was 0.24, and the PA index was 0.47. These results seem to indicate that the interaction between psychological demands and HCC follows the vantage sensitivity model.

**Figure 3:**
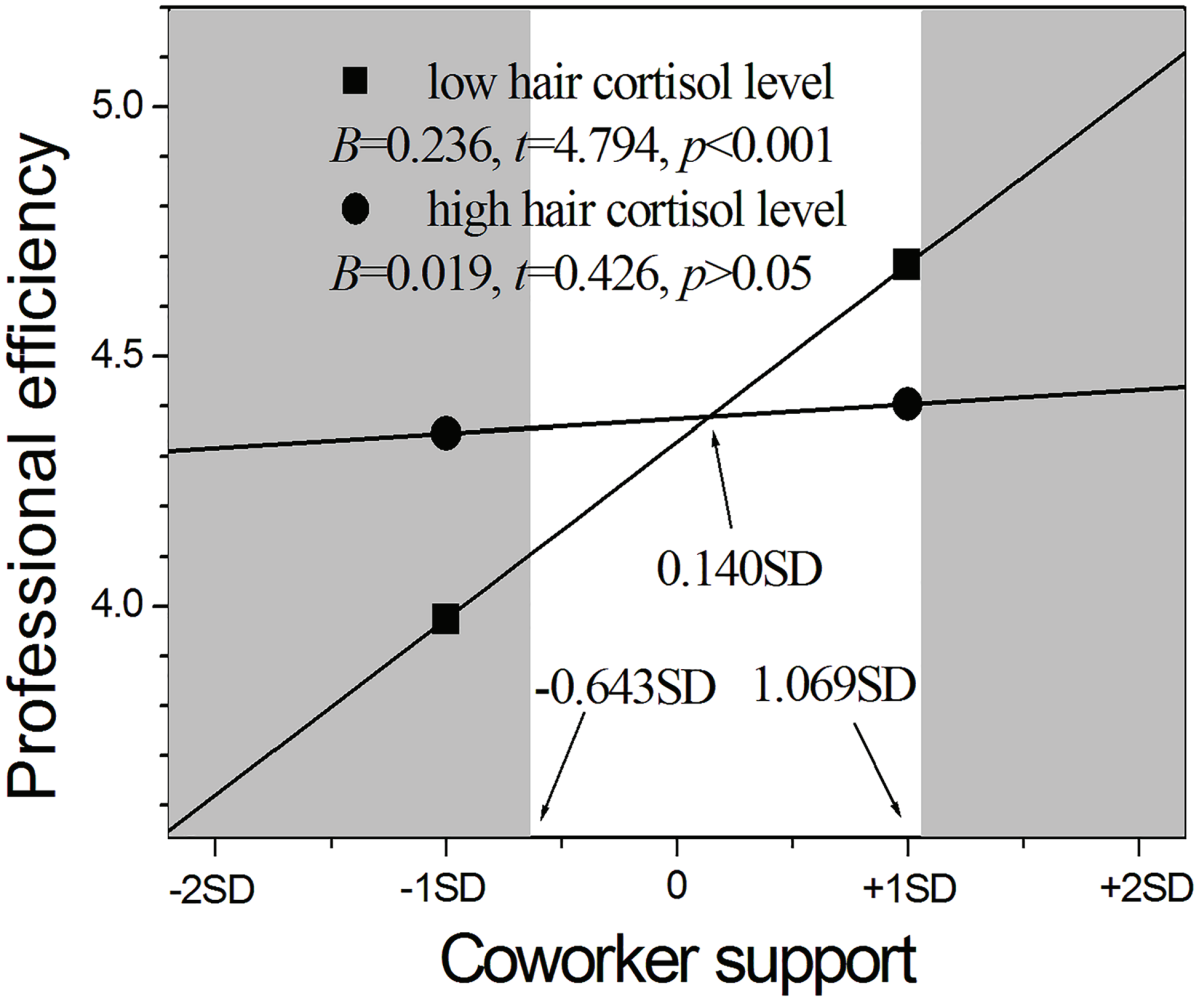
The moderating role of hair cortisol in the relationship between coworker support and professional efficiency.

As shown in Figure 3, the stronger influence of coworker support on professional efficiency existed in nurses with low cortisol levels (*B* = 0.236, *p* < 0.001) than those with high cortisol levels (*B* = 0.019, *p* > 0.05). The RoS results further revealed that nurses with low cortisol levels had significantly higher professional efficiency than those with high cortisol levels in the context of higher coworker support above 1.069 SD from the mean (see the right shaded area in Figure 3) and had significantly lower professional efficiency in the context of lower coworker support below −0.643 SD from the mean (see the left shaded area in Figure 3), but the two groups had no difference in the region between −0.643 SD and 1.069 SD from the mean of coworker support. The PoI index was 0.43, and the PA index was 0.42. These results indicated that the interaction between coworker support and HCC supports the differential susceptibility model.

## Discussion

This study confirmed that the interaction patterns of stress reactivity with job control and coworker support among Chinese hospital nurses follow the differential susceptibility model and that the interaction between stress reactivity and psychological demands supports the vantage sensitivity model. These results extended the interaction pattern between job resources and work environments from the traditional perspective of the diathesis stress model to the novel perspectives of the differential susceptibility model and the vantage sensitivity model when the focus was expanded from the adverse and pathogenic responses in adverse environments to the adaptive responses in supportive environments. These results also provided another evidence for the extension of the JDR model to personal biological resources from social and organizational resources and personal mental resources, together with a recent finding that the interaction patterns between stress reactivity and emotional labor might follow the differential susceptibility model or the vantage sensitivity model (Deng et al., 2020).

In predicting nurses’ professional efficiency, both job control and coworker support showed the same interaction patterns with stress reactivity, which was consistent with the differential susceptibility model. Specifically, nurses with high hair cortisol levels had significantly higher professional efficiency than those with low cortisol levels in the context of high job control and had significantly lower professional efficiency in the context of low job control. Nurses with low cortisol levels had significantly higher professional efficiency than those with high cortisol levels in the context of high coworker support and had significantly lower professional efficiency in the context of low coworker support. These results indicated that for nurses’ professional efficiency, high hair cortisol level was the plasticity factor in the context of job control, i.e., a high hair cortisol level was a promoting factor in the context of high job control and a risk factor in low job control. Whereas low hair cortisol level was the plasticity factor in the context of coworker support, i.e., a low hair cortisol level was a promoting factor in more coworker support and a risk factor in the context of less coworker support. It was apparent that stress reactivity showed the moderating role differing between job control and coworker support in their association with professional efficiency. As mentioned in the *Introduction*, stress reactivity is a highly biological sensitivity to context (Boyce and Ellis, 2005). Thus, compared with those with a low stress reactivity, individuals with a high stress reactivity were possibly more sensitive to the stressful context related to job demands in workplace environments, thereby being in the allostasis with high frequency according to allostatic load theory (McEwen, 2003), and being highly susceptible to the environmental factors in the workplace. Indeed, nurses with a high stress reactivity received stronger positive influence from job control, showing high professional efficiency with high job controllability and low professional efficiency with low job controllability. On the other hand, because of their high sensitivity to stressful context in the workplace, they would be partial to put more physical and mental resources in coping with primary job stress from highly stressful job demands (e.g., job uncontrollability and psychological demands). Thus, they would put fewer resources in coping with secondary job demands in other aspects of job characteristics (e.g., coworker support) according to the conversation of resources model (Hobfoll, 1998), thereby be relatively insensitive to secondary job demands. As a result, they received weaker influence from coworker support. In contrast, because of their relatively low sensitivity to job stress, nurses with a low stress reactivity would consume fewer resources in coping with highly stressful job demands, thereby keeping more resources to put into other job demands. Therefore, they received stronger positive influence from coworker support, showing high professional efficiency with high coworker support and low professional efficiency with low coworker support.

Except for the three interactions mentioned above, the other interactions between stress reactivity and job characteristics were not significant. This might be because there is a mismatch among stress reactivity, other job characteristics, and job burnouts in the emotional, cognitive, and biological levels. For example, hair cortisol was not related to job characteristics and job burnouts in this study, although hair cortisol is proven to be closely associated with emotional, spiritual, and sociopsychological stress in a previous study (Russell et al., 2012). Hair cortisol as a biomarker of stress reactivity is considered to mainly reflect personal resources at the biological level (Boyce and Ellis, 2005). As discussed above, three subscales of MBI burnouts give the fatigue and exhaustion syndromes at the emotional, cognitive, and behavioral level (Schutte et al., 2000), respectively. Psychological demands, job control, supervisor support, and coworker support are psychological and social characteristics of jobs (Karasek, 1985). The explanation needs to be examined in future research.

### Practical Implications

The role of stress reactivity in moderating the relationships between job characteristics and job burnouts gave practical implication that managers should consider the interindividual differences in personal biological resources (e.g., stress reactivity) and make the differential protocol when they provide interventions that improve nurses’ efficacy in dealing with their job-related demands and shaping nurses’ outcomes. For example, this study found that high stress reactivity was the plasticity factor for professional efficiency in the context of job control, but low stress reactivity was the plasticity factor in the context of coworker support. In order to improve nurses’ professional efficiency, managers should provide the position with high job controllability for individuals with a high stress reactivity but provide high coworker support for individuals with a low stress reactivity. Similarly, low stress reactivity as the vantage sensitivity characteristic of emotional exhaustion in the context of psychological demands implied that managers should weaken psychological demands for individuals with a high stress reactivity in order to reduce nurses’ emotional exhaustion.

### Strength, Limitations, and Future Research

The strength in the current study was to utilize hair cortisol level as a biomarker of stress reactivity in 1 month rather than the traditional biomarkers, such as salivary cortisol level. Hair cortisol level is a biomarker more reliably assessing the long-term activity of the HPA axis or stress reactivity (Russell et al., 2012) than urinary and salivary cortisol levels reflecting the acute and short-term activity of the HPA axis over several hours and up to 1 day (Dickerson and Kemeny, 2004). Moreover, it shows high consistency with the average level of multiple-day salivary cortisol levels within 1 month (Zhang et al., 2018; Chen et al., 2019). Additionally, cortisol level in 1-cm hair segment could better match the time span of the psychological states including job characteristics and job burnouts over the past 1 month if the hair growth rate is 1 cm per month.

The current study has several limitations. Firstly, the current cross-sectional design limited the understanding of the causality between variables, although job characteristics were hypothesized as independent variables, hair cortisol level as a moderator, and job burnouts as dependent variables. The future research should be based on a longitudinal study for examining causal relationships. Secondly, this study utilized only hair cortisol level as the biomarker of long-term stress reactivity. In the future, other biomarkers of stress reactivity would be worthy of consideration for the generalization of stress reactivity as job resources at the personal biological level. Thirdly, this study did not examine other job demands, such as physical demands, task complexity, and job security. Finally, this study was based on a homogeneous sample, Han Chinese female nurses in city hospitals, China. In short, for the generalization of the results, future research should cover more job characteristics and be conducted on a heterogeneous sample including other occupations and other ethnic groups in China and other countries.

## Conclusion

Stress reactivity could moderate associations of psychological demands, job control, and coworker support with job burnouts among Chinese hospital nurses. Specifically, the interaction of HCC with job control could positively predict professional efficiency, with psychological demands could negatively predict emotional exhaustion, and with coworker support could negatively predict professional efficiency. The interaction patterns of stress reactivity with both job control and coworker support were consistent with the differential susceptibility model, but the interaction between stress reactivity and psychological demands supported the vantage sensitivity model. For nurses’ professional efficiency, high stress reactivity was the plasticity factor in the context of job control, but low stress reactivity was the plasticity factor in the context of coworker support. For emotional exhaustion, low stress reactivity was the vantage sensitivity characteristic in the context of psychological demands. These results would be helpful in understanding more fully the importance of biological processes that interact with the work environmental influences in employees’ dealing with job-related demands and shaping employees’ outcomes.

## Data Availability Statement

The raw data supporting the conclusions of this article will be made available by the authors, without undue reservation.

## Ethics Statement

The studies involving human participants were reviewed and approved by the Health Science Research Ethics Board of Southeast University. The patients/participants provided their written informed consent to participate in this study.

## Author Contributions

All authors had substantial intellectual contributions to this study as follows. HD contributed to the conceptualization, data analysis, writing and editing, and funding acquisition. YZ, HW, and YL contributed to the data analysis and writing and editing. XQ contributed to the conceptualization, data collection, and funding acquisition. JL contributed to the methodology and funding acquisition. CJ contributed to the data collection and funding acquisition. All authors contributed to the article and approved the submitted version.

### Conflict of Interest

The authors declare that the research was conducted in the absence of any commercial or financial relationships that could be construed as a potential conflict of interest.

## References

[ref1] Aiken L. S. West S. G. (1991). Multiple regression: testing and interpreting interactions. Eval. Pract. 45, 119–120. 10.1057/jors.1994.16

[ref2] Bakker A. B. Demerouti E. (2007). The Job Demands-Resources model: state of the art. J. Manag. Psychol. 22, 309–328. 10.1108/02683940710733115

[ref3] Bakker A. B. Demerouti E. Euwema M. C. (2005). Job resources buffer the impact of job demands on burnout. J. Occup. Health Psychol. 10, 170–180. 10.1037/1076-8998.10.2.170, PMID: 15826226

[ref4] Bakker A. B. Demerouti E. Taris T. W. Schaufeli W. B. Schreurs P. J. G. (2003). A multigroup analysis of the job demands-resources model in four home care organizations. Int. J. Stress. Manag. 10, 16–38. 10.1037/1072-5245.10.1.16

[ref5] Belsky J. Bakermans-Kranenburg M. J. Van Ijzendoorn M. H. (2007). For better and for worse: differential susceptibility to environmental influences. Curr. Dir. Psychol. Sci. 16, 300–304. 10.1111/j.1467-8721.2007.00525.x

[ref6] Belsky J. Pluess M. (2009). Beyond diathesis stress: differential susceptibility to environmental influences. Psychol. Bull. 135, 885–908. 10.1037/a0017376, PMID: 19883141

[ref7] Boyce W. T. Ellis B. J. (2005). Biological sensitivity to context: I. An evolutionary-developmental theory of the origins and functions of stress reactivity. Dev. Psychopathol. 17:271. 10.1017/s0954579405050145, PMID: 16761546

[ref8] Chen Z. Zhang Q. Chen S. Wang W. Deng H. (2019). Determination, intercorrelation and intraindividual stability of five steroids in hair, saliva and urine among Chinese college students. Steroids 149:108418. 10.1016/j.steroids.2019.05.010, PMID: 31150683

[ref9] Cheng Y. Luh W. M. Guo Y. L. (2003). Reliability and validity of the Chinese version of the Job Content Questionnaire in Taiwanese workers. Int. J. Behav. Med. 10, 15–30. 10.1207/S15327558IJBM1001_02, PMID: 12581945

[ref10] Chi N. W. Grandey A. A. Diamond J. A. Krimmel K. R. (2011). Want a tip? Service performance as a function of emotion regulation and extraversion. J. Appl. Psychol. 96, 1337–1346. 10.1037/a0022884, PMID: 21480687

[ref11] Demerouti E. Bakker A. B. Nachreiner F. Schaufeli W. B. (2001). The job demands-resources model of burnout. J. Appl. Psychol. 86, 499–512. 10.1037/0021-9010.86.3.499, PMID: 11419809

[ref12] Deng H. Wu H. Qi X. Jin C. Li J. (2020). Stress reactivity influences the relationship between emotional labor strategies and job burnouts among Chinese hospital nurses. Neural Plast. 2020, 1–13. 10.1155/2020/8837024, PMID: 33029118 PMC7528115

[ref13] Dickerson S. S. Kemeny M. E. (2004). Acute stressors and cortisol responses: a theoretical integration and synthesis of laboratory research. Psychol. Bull. 130, 355–391. 10.1037/0033-2909.130.3.355, PMID: 15122924

[ref14] Diestel S. Rivkin W. Schmidt K. H. (2015). Sleep quality and self-control capacity as protective resources in the daily emotional labor process: results from two diary studies. J. Appl. Psychol. 100, 809–827. 10.1037/a0038373, PMID: 25486259

[ref15] Ellis B. J. Boyce W. T. Belsky J. Bakermans-Kranenburg M. J. Van Ijzendoorn M. H. (2011). Differential susceptibility to the environment: an evolutionary–neurodevelopmental theory. Dev. Psychopathol. 23, 7–28. 10.1017/S0954579410000611, PMID: 21262036

[ref49] Fox J. (1991). Regression Diagnostics: An Introduction. Newbury Park, CA: Sage.

[ref16] Gu Y. You X. (2019). Recovery experiences buffer against adverse well-being effects of workplace surface acting: a two-wave study of hospital nurses. J. Adv. Nurs. 76, 209–220. 10.1111/jan.14236, PMID: 31612517

[ref17] Halbesleben J. R. B. Buckley M. R. (2004). Burnout in organizational life. J. Manag. 30, 859–879. 10.1016/j.jm.2004.06.004

[ref18] Hayes A. F. Matthes J. (2009). Computational procedures for probing interactions in OLS and logistic regression: SPSS and SAS implementations. Behav. Res. Methods 41, 924–936. 10.3758/BRM.41.3.924, PMID: 19587209

[ref19] Hobfoll S. E. (1998). Stress, culture, and community: the psychology and philosophy of stress. Br. J. Med. Psychol. 31, 254–262. 10.1037/h0087094

[ref20] Hobfoll S. E. (2002). Social and psychological resources and adaptation. Rev. Gen. Psychol. 6, 307–324. 10.1037/1089-2680.6.4.307

[ref21] Hobfoll S. E. Johnson R. J. Ennis N. Jackson A. P. (2003). Resource loss, resource gain, and emotional outcomes among inner city women. J. Pers. Soc. Psychol. 84, 632–643. 10.1037/0022-3514.84.3.632, PMID: 12635922

[ref22] Jex S. M. Elacqua T. C. (1999). Self-esteem as a moderator: a comparison of global and organization-based measures. J. Occup. Organ. Psychol. 72, 71–81. 10.1348/096317999166509

[ref23] Johnson J. V. Hall E. M. (1988). Job strain, work place social support, and cardiovascular-disease-a cross-sectioonal study of a random sample of the Swedish working population. Am. J. Public Health 78, 1336–1342. 10.2105/AJPH.78.10.1336, PMID: 3421392 PMC1349434

[ref24] Johnson H. A. M. Spector P. E. (2007). Service with a smile: do emotional intelligence, gender and autonomy moderate the emotional labor process? J. Occup. Health Psychol. 12, 319–333. 10.1037/1076-8998.12.4.319, PMID: 17953492

[ref25] Karasek R. A. (1979). Job demands, job decision latitude, and mental strain-Implications for job redesign. Adm. Sci. Q. 24, 285–308. 10.2307/2392498

[ref26] Karasek R. (1985). Job Content Instrument Questionnaire and User’s Guide, Version 1.1. Department of Industrial and Systems Engineering. University of Southern California.

[ref27] Karasek R. Brisson C. Kawakami N. Houtman I. Bongers P. Amick B. (1998). The Job Content Questionnaire (JCQ): an instrument for internationally comparative assessments of psychosocial job characteristics. J. Occup. Health Psychol. 3, 322–355. 10.1037/1076-8998.3.4.322, PMID: 9805280

[ref28] Kristensen T. S. Borritz M. Christensen E. V. B. K. (2005). The Copenhagen Burnout Inventory: a new tool for the assessment of burnout. Work Stress. 19, 192–207. 10.1080/02678370500297720

[ref29] LeBeau M. A. Montgomery M. A. Brewer J. D. (2011). The role of variations in growth rate and sample collection on interpreting results of segmental analyses of hair. Forensic Sci. Int. 210, 110–116. 10.1016/j.forsciint.2011.02.015, PMID: 21382678

[ref30] Li C. Shi K. (2003). The influence of distributive justice and procedural justice on job burnout. Acta Psychol. Sin. 35, 677–684. 10.1023/A:1022289509702

[ref31] McEwen B. S. (2003). Mood disorders and medical illness: mood disorders and allostatic load. Biol. Psychiatry 54, 200–207. 10.1016/S0006-3223(03)00177-X, PMID: 12893096

[ref32] Mkikangas A. Kinnunen U. (2003). Psychosocial work stressors and well-being: self-esteem and optimism as moderators in a one-year longitudinal sample. Personal. Individ. Differ. 35, 537–557. 10.1016/S0191-8869(02)00217-9

[ref33] Obradovi J. Bush N. R. Stamperdahl J. Adler N. E. Boyce W. T. (2010). Biological sensitivity to context: the interactive effects of stress reactivity and family adversity on socioemotional behavior and school readiness. Child Dev. 81, 270–289. 10.1111/j.1467-8624.2009.01394.x, PMID: 20331667 PMC2846098

[ref34] Obradovi J. Portilla X. A. Ballard P. J. (2015). Biological sensitivity to family income: differential effects on early executive functioning. Child Dev. 87, 374–384. 10.1111/cdev.12475, PMID: 26709089

[ref35] Owens S. A. Helms S. W. Rudolph K. D. Hastings P. D. Nock M. K. Prinstein M. J. (2018). Interpersonal stress severity longitudinally predicts adolescent girls’ depressive symptoms: the moderating role of subjective and HPA Axis stress responses. J. Abnorm. Child Psychol. 47, 895–905. 10.1007/s10802-018-0483-x, PMID: 30298267 PMC6541026

[ref36] Pluess M. Belsky J. (2013). Vantage sensitivity: individual differences in response to positive experiences. Psychol. Bull. 139, 901–916. 10.1037/a0030196, PMID: 23025924

[ref37] Podsakoff P. M. MacKenzie S. B. Lee J.-Y. Podsakoff N. P. (2003). Common method biases in behavioral research: a critical review of the literature and recommended remedies. J. Appl. Psychol. 88, 879–903. 10.1037/0021-9010.88.5.879, PMID: 14516251

[ref38] Pragst F. Balikova M. A. (2006). State of the art in hair analysis for detection of drug and alcohol abuse. Clin. Chim. Acta 370, 17–49. 10.1016/j.cca.2006.02.019, PMID: 16624267

[ref39] Pugh S. D. Groth M. Hennig-Thurau T. (2011). Willing and able to fake emotions: a closer examination of the link between emotional dissonance and employee well-being. J. Appl. Psychol. 96, 377–390. 10.1037/a0021395, PMID: 21058805

[ref40] Roisman G. I. Newman D. A. Fraley R. C. Haltigan J. D. Haydon K. C. (2012). Distinguishing differential susceptibility from diathesis–stress: recommendations for evaluating interaction effects. Dev. Psychopathol. 24, 389–409. 10.1017/S0954579412000065, PMID: 22559121

[ref41] Russell E. Koren G. Rieder M. Van Uum S. (2012). Hair cortisol as a biological marker of chronic stress: current status, future directions and unanswered questions. Psychoneuroendocrinology 37, 589–601. 10.1016/j.psyneuen.2011.09.009, PMID: 21974976

[ref42] Salvagioni D. A. J. Melanda F. N. Mesas A. E. González A. D. Andrade S. M. D. (2017). Physical, psychological and occupational consequences of job burnout: a systematic review of prospective studies. PLoS One 12:e0185781. 10.1371/journal.pone.0185781, PMID: 28977041 PMC5627926

[ref43] Schafer J. L. Graham J. W. (2002). Missing data: our view of the state of the art. Psychol. Methods 7, 147–177. 10.1037/1082-989X.7.2.147, PMID: 12090408

[ref44] Schaufeli W. Leiter M. Maslach C. Jackson S. (1996). Maslach Burnout Inventory -- General Survey (GS). Palo Alto, CA: Consulting Psychologists Press.

[ref45] Schreurs B. Guenter H. Hülsheger U. van Emmerik H. (2014). The role of punishment and reward sensitivity in the emotional labor process: a within-person perspective. J. Occup. Health Psychol. 19, 108–121. 10.1037/a0035067, PMID: 24447225

[ref46] Schutte N. Toppinen S. Kalimo R. Schaufeli W. (2000). The factorial validity of the Maslach Burnout Inventory-General Survey (MBI-GS) across occupational groups and nations. J. Occup. Organ. Psychol. 73, 53–66. 10.1348/096317900166877

[ref47] Spiga F. Walker J. J. Terry J. R. Lightman S. L. (2014). HPA axis-rhythms. Compr. Physiol. 4, 1273–1298. 10.1002/cphy.c140003, PMID: 24944037

[ref48] Tremblay M. A. Messervey D. (2011). The Job Demands-Resources model: Further evidence for the buffering effect of personal resources. SAJIP 37, 876–886. 10.4102/sajip.v37i2.876

[ref50] Usman M. Ali M. Yousaf Z. Anwar F. Waqas M. Khan M. A. S. (2020). The relationship between laissez-faire leadership and burnout: Mediation through work alienation and the moderating role of political skill. Can. J. Adm. Sci. 37, 423–434. 10.1002/cjas.1568

[ref51] van der Doef M. Maes S. (1998). The Job Demand Control (-Support) model and physical health outcomes: a review of the strain and buffer hypothesis. Psychol. Health 13, 909–936. 10.1080/08870449808407440

[ref52] Van Yperen N. W. Snijders T. A. B. (2000). A multilevel analysis of the demands–control model: Is stress at work determined by factors at the group level or the individual level? J. Occup. Health Psychol. 5, 182–190. 10.1037/1076-8998.5.1.182, PMID: 10658895

[ref53] Wiel N. M. H. Goozen S. H. Matthys W. Snoek H. Engeland H. V. (2004). Cortisol and treatment effect in children with disruptive behavior disorders: a preliminary study. J. Am. Acad. Child Adolesc. Psychiatry. 43, 1011–1018. 10.1097/01.chi.0000126976.56955.43, PMID: 15266196

[ref54] Wosu A. C. Valdimarsdóttir U. Shields A. E. Williams D. R. Williams M. A. (2013). Correlates of cortisol in human hair: implications for epidemiologic studies on health effects of chronic stress. Ann. Epidemiol. 23, 797.e792–811.e792. 10.1016/j.annepidem.2013.09.006, PMID: 24184029 PMC3963409

[ref55] Wu X. J. Li J. Q. Liu G. Liu Y. Cao J. Jia Z. X. (2018). The effects of emotional labor and competency on job satisfaction in nurses of China: a nationwide cross-sectional survey. Int. J. Nurs. Sci. 5, 383–389. 10.1016/j.ijnss.2018.08.001, PMID: 31406852 PMC6626301

[ref56] Xanthopoulou D. Bakker A. B. Demerouti E. Schaufeli W. B. (2007). The role of personal resources in the job demands-resources model. Int. J. Stress. Manag. 14, 121–141. 10.1037/1072-5245.14.2.121

[ref57] Xie J. L. (1996). Karasek’s model in the People’s Republic of China: effects of job demands, control, and individual differences. Acad. Manag. J. 39, 1594–1618. 10.2307/257070

[ref58] Xu Y. Liu Y. Chen Z. Zhang J. Deng H. Gu J. (2019). Interaction effects of life events and hair cortisol on perceived stress, anxiety, and depressive symptoms among Chinese adolescents: testing the differential susceptibility and diathesis-stress models. Front. Psychol. 10:297. 10.3389/fpsyg.2019.00297, PMID: 30890975 PMC6411789

[ref59] Zhang Q. Chen Z. Chen S. Yu T. Wang J. Wang W.. (2018). Correlations of hair level with salivary level in cortisol and cortisone. Life Sci. 193, 57–63. 10.1016/j.lfs.2017.11.037, PMID: 29180003

